# Ventricular Dysfunction: Tachycardia induced Cardiomyopathy

**Published:** 2008-05-01

**Authors:** V Ramesh Iyer

**Affiliations:** Assistant Professor of Pediatrics, University of Connecticut School of Medicine, Division of Cardiology, Connecticut Children's Medical Center, Hartford, CT 06106, U.S.A

**Keywords:** Arrhythmia, Tachycardia induced cardiomyopathy, ventricular dysfunction

## Abstract

Tachycardia induced cardiomyopathy (TIC) is defined as atrial or ventricular dysfunction as a result of prolonged elevated heart rate that is reversible upon control of the arrhythmia. There may be no underlying structural heart disease primarily responsible for the cardiac dysfunction. We present some examples of arrhythmias causing TIC and the resolution of the ventricular dysfunction following their appropriate management. Included in the review are also the pathophysiology, clinical spectrum, diagnosis and appropriate management of the condition.

## Introduction

Tachycardia induced cardiomyopathy (TIC) occurs as a result of prolonged elevated heart rates and resolves with treatment of the arrhythmia [1,2]. Different arrhythmias have been reported to result in ventricular dysfunction. These include automatic atrial arrhythmias including ectopic atrial tachycardia, atrial fibrillation and re-entry atrial and atrioventricular arrhythmias such as atrial flutter [3] and intra atrial re-entry tachycardia (IART) in congenital heart disease (CHD) and permanent junctional reciprocating tachycardia (PJRT) [4] and incessant slow atrioventricular nodal tachycardia (AVNRT) and atrioventricular (AV) re-entry tachycardias [5]. Ventricular arrhythmias such as sustained ventricular tachycardia (VT) [6] and premature ventricular contractions (PVC) [7] have also been reported to cause ventricular dilation and dysfunction. The diagnosis of TIC needs to considered when no other obvious explanation is forthcoming for cardiac dysfunction and is often retrospective as the patient improves clinically with rate and/or rhythm control. The prevalence is hard to estimate as it is mainly described in case reports. In some cases it may be difficult to determine whether the tachycardia is secondary to underlying cardiomyopathy or is playing a causative role (especially in arrhythmias such as atrial fibrillation [8])

## Case report 1

**Figure 1:**
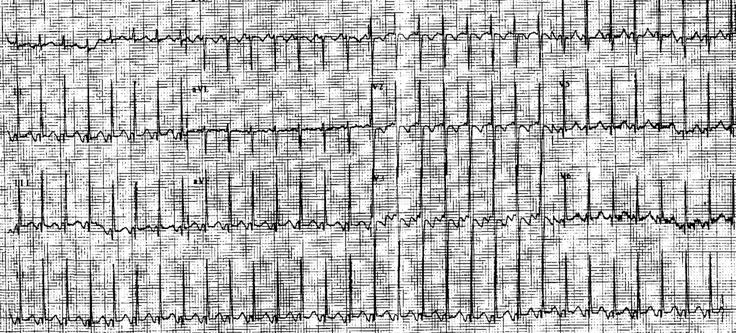
6 month old with PJRT who has narrow complex tachycardia with long RP interval (260ms) and negative P waves in leads II, III, AVF.

A 6 month old infant girl presents with a 5-6 day history of poor feeding and tachypnea. She looked pale and lethargic. Examination revealed elevated heart rate of 180 beats per minute (BPM) with gallop. EKG showed (Figure 1) narrow complex tachycardia with a long RP interval and negative P waves in leads II, III, and AVF consistent with PJRT. Echocardiogram showed depressed ventricular function with no structural abnormalities. Management included drug therapy with Flecanide and Sotalol with normalization of EKG and improvement in ventricular function in one week.

## Case report 2

**Figure 2:**
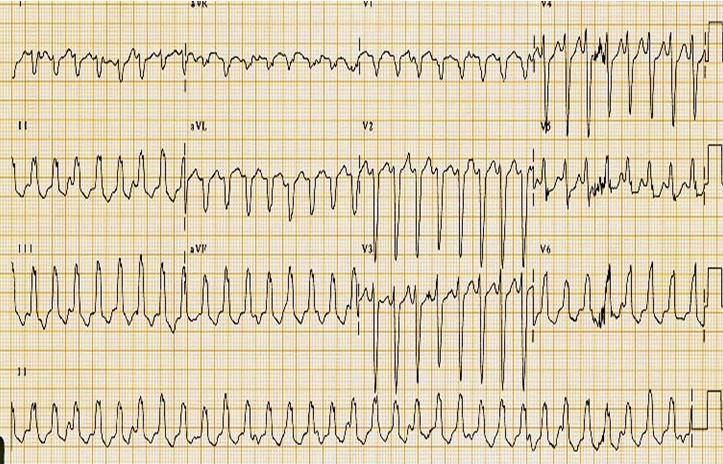
6 yr old with sustained RV outflow tract VT at a rate of 180 bpm. The EKG also shows VA dissociation.

**Figure 3:**
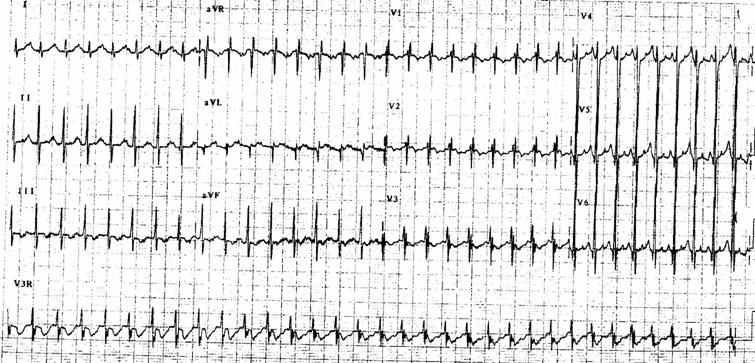
EKG showing ectopic atrial tachycardia (right atrial crista) at rate of 200bpm. P waves are positive in lead I and AVF and negative in V1.

6 yr old boy presented to the emergency department with a 4-day history of fatigue. Evaluation revealed a pale child, 23kg, with a heart rate of 182 bpm. EKG revealed wide complex tachycardia (QRS 128ms, 181bpm) with VA dissociation (Figure 2), LBBB, and inferior QRS axis (45º). CXR showed cardiomegaly (Figure 3). Echocardiogram showed moderate to severe biventricular dysfunction with moderate mitral valve insufficiency. The fractional shortening was 15% with an ejection fraction of 28%. VT did not respond to intravenous (IV) lidocaine but converted to sinus rhythm with Procainamide bolus of 5mg/kg. Ventricular function improved and the patient was taken to the EP laboratory for a successful ablation of a right ventricular (RV) outflow tract focus.

## Pathophysiology

Several pathological changes have been described but it remains unclear as to whether they play an etiologic role or they arise as a consequence of the tachycardia. Hemodynamic alterations include reduced cardiac output, biventricular dysfunction, elevated filling pressures and elevated systemic vascular resistance [9]. There is also associated neurohormonal activation characterized by elevated plasma atrial natriuretic peptide levels, high epinephrine and nor-epinephrine concentration, increased renin and aldosterone activity [10]. The exact mechanisms are not well understood. Some of the changes that have been described in animal and human studies are as follows:

### Myocardial ischemia and energy store depletion

High energy myocardial stores have been shown to get depleted in animal models with persistent tachycardia. These include diminished Na-K-ATPase activity and lower myocardial levels of adenosine triphosphate (ATP), phosphocreatine and creatine [11,12]. They may result from increased activity of Krebs cycle enzymes and mitochondrial injury [13]. These changes have been reproducible and reversible in ischemic myocardial injury [14]. This may explain a cause-effect relationship between TIC and myocardial ischemia.

### Abnormal receptor response and oxidative stress

There is decreased beta-adrenergic responsiveness [15] and abnormal Calcium (Ca) channel activity and sarcoplasmic reticulum Ca response resulting in reduced cellular energy described in different animal models [12,16]. There may also be down regulation of myocyte beta-1 receptor density. Oxidative stress injury of the myocardium may result in accumulation of peroxynitrite and subsequent modification of myofibrillar proteins and energetics. This results in an imbalance of pro-oxidant and anti-oxidant pathways [17].

### Genetic basis / ACE gene polymorphism

Elevated levels of angiotensin converting enzyme (ACE) are associated with deletion of the ACE gene (D). The DD (deletion) phenotype has been shown to be significantly more common in patients with TIC [18] (they also have elevated serum ACE levels).

### Histologic Modifications

The various histological changes associated with TIC include myocyte hyperplasia and lengthening [19], myocardial fibrosis, impaired coronary reserve and apoptosis.

## Clinical Manifestation

Clinical symptoms in children may be non specific with symptoms of shortness of breath, fatigue, exercise intolerance and palpitations. Infants often present with poor feeding and tachypnea. The risk of sudden death may be as high as 10 % [20]. Physical examination may reveal a tachycardia out of proportion to age with or without edema, poor skin perfusion, gallop rhythm and regurgitant murmurs from AV valve insufficiency. Other signs of CHF such as elevated jugular venous pulse, liver enlargement and basal rales may also be present.

## Differential Diagnosis

The diagnosis of TIC requires a high index of suspicion. Workup includes an EKG that may show the culprit arrhythmia responsible. Chest X-ray is helpful in confirming an enlarged cardiac silhouette and increased pulmonary vascular markings indicating elevated filling pressures. Echocardiogram often reveals LV and LA dilation with diminished shortening and ejection fractions. There may be evidence of systolic and / or diastolic ventricular dysfunction. Differential diagnosis includes other causes of cardiomyopathy including dilated, infective and ischemic etiologies. Complete or partial recovery of cardiac function with rate or rhythm control favors TIC.

## Arrhythmias causing TIC

Almost all arrhythmias can cause TIC. Some of the common once are listed in Table 1.

### Supraventricular arrhythmias

In newborns re-entry SVT is the commonest cause of TIC. Some ventricular dysfunction is almost universal as diagnosis is often delayed. SVT at higher rates is better tolerated in this age group and infants often present after days of onset of the arrhythmia [21]. Longer the duration of SVT, worse the cardiac function. The prognosis and recovery of cardiac function after control of SVT is excellent.

Ectopic atrial tachycardia [22] is probably the second most common cause of TIC especially in older children (Figure 3). They can arise from either the RA or LA. Most EATs have no specific etiology but infection has been suspected in some cases. Post operative (both acute and chronic) states have known to cause ectopic foci that can be responsible. Clinical spectrum is similar and the duration and the rate of the tachycardia determine the severity of TIC. Radiofrequency or cryo ablation is usually the treatment of choice in older children after initial supportive management of the cardiac dysfunction which may include diuretics (frusemide). Medical antiarrhythmia management with flecanide, procainamide, sotalol or amiodarone (or combination therapy) is usually successful.

PJRT is mediated by a slow retrograde conducting concealed accessory pathway which is usually located in the posteroseptal right atrium. The SVT has a typical long RP interval with negative P waves in leads II, III, and AVF (Figure 1). The SVT can be refractory to medical management. They respond well to ablative therapy [4]. Anti arrhythmic drug therapy may include various combinations of flecanide, sotalol and amiodarone.

Atrial flutter and intra-atrial re-entry tachycardia are more common in children with CHD who have had surgical interventions and subsequent atrial scarring. Multiple atrial re-entry circuits may be present and ablation with or without antiarrhythmic drug therapy may be effective. Cardiac dysfunction is usually reversible with control of atrial flutter [3]. Thromboembolic risk is also increased and has to be taken into consideration after diagnosis before treatment plans are formulated.

Postoperative junctional ectopic tachycardia (JET) has been seen with surgical repair of VSDs, tetralogy of Fallot and AV canal defects. The congenital form of JET occurs in non surgical cases and may be inherited [23]. These tachycardias gradually warm up and can cause cardiac dysfunction over a period of few hours after surgical intervention. Medical management includes appropriate timely recognition and use of antiarrhythmic agents such as amiodarone with control of body temperature. Congenital JET is rare but more difficult to recognize and treat. Multiple drugs may need to be used including amiodarone, sotalol and flecanide.

Atrial fibrillation is uncommon in children but a common cause of TIC in the adult population and may depress cardiac function by 15-20% [8].

### Ventricular Arrhythmias

Both non-sustained and sustained ventricular arrhythmia can result in cardiac dysfunction. Frequent RV outflow tract PVCs (LBBB morphology with inferior axis) has been described by authors [6] with improvement of symptoms and function after successful ablative treatment. Sustained VTs such as bundle branch re-entry VTs are more common in structural heart disease (such as primary cardiomyopathy) and cause-effect relationship may be confusing. Other VTs such scar VTs (after CHD repair) and idiopathic LV VT may compromise ventricular function in the acute phase and improve after rhythm control. Ventricular pacing at fast rates has also been associated with depressed cardiac function.

## Prognosis

The prognosis depends on the rate and duration of the arrhythmias which are two factors that influence severity of cardiac dysfunction. Younger children (especially newborns and infants) tend to make complete recovery. Recurrence of the arrhythmia after apparent resolution may be associated with precipitous decline in LV systolic function and rapid development of symptomatic heart failure [20]. Diagnosis of TIC may be difficult in some cases where the culprit arrhythmia is relatively slow and not incessant and may be retrospective with improvement of cardiac function after rate and/or rhythm control. Long term treatment may include diuretics (such as furesemide), ACE inhibitors, betablockers with adjunctive RF ablation or antiarrhythmic drugs. Improvement of ventricular function occurs as early as one week and complete resolution in 4-6 weeks.

## Conclusion

The recognition of TIC with arrhythmias in children is important as appropriate treatment (rhythm and/or rate control) has a good outcome. TIC may be one the one of the most common unrecognized curable cause of heart failure that needs to taken into consideration in the differential diagnosis of idiopathic dilated cardiomyopathy.

## Figures and Tables

**Table 1:**
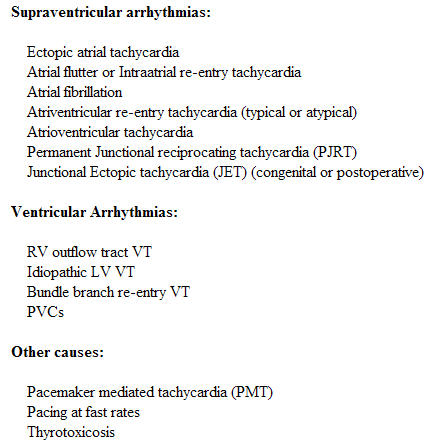
Arrhythmias causing tachycardiomyopathy
